# Protein Decoys, Alcohol, and Energy Intake: Testing a Mechanistic‐Ecological Model

**DOI:** 10.1111/obr.70138

**Published:** 2026-05-19

**Authors:** Amanda Grech, Stephen J. Simpson, David Raubenheimer

**Affiliations:** ^1^ Charles Perkins Centre University of Sydney NSW Australia; ^2^ School of Life and Environmental Science University of Sydney Australia

**Keywords:** alcohol, energy balance, protein

## Abstract

Ultra‐processed diets are associated with excessive energy intake, but studies examining the role of alcohol have produced conflicting results, despite the high energy density of ethanol. We constructed a mechanistic‐ecological model to explain this inconsistency and tested its predictions using population dietary data. The model builds on experimentally established mechanisms to predict that, whereas alcohol will contribute ethanol calories irrespective of diet, its impact on total macronutrient energy intake varies depending on dietary pattern. Alcohol consumption stimulates FGF21, a hormone that increases savory (umami) preference and reduces sweet preference. In minimally processed food environments—where umami flavor is a relatively reliable indicator of protein in foods—umami‐seeking should direct consumers toward satiating, high‐protein foods, with limited effect on macronutrient energy intake. In contrast, on diets rich in ultra‐processed savory foods and/or high‐fat unprocessed meats, the correlation between umami flavor characteristics and protein is broken by umami‐flavored low‐protein foods (protein decoys), so alcohol instead drives dietary protein dilution and, through attenuated protein satiation, increased food and macronutrient energy intake (protein leverage). Mixture analysis paired with nutritional geometry confirmed these predictions in population data, suggesting the model may generalize to ecological contexts. Our results potentially explain why alcohol has variable effects on energy balance across dietary patterns and suggest that it exacerbates the obesogenic effects of ultra‐processed diets—a potentially important interaction given their global rise.

## 1 Introduction

Ultra‐processed foods (UPF) and alcohol are both modifiable risk factors for noncommunicable diseases. Strong evidence from experimental [1, 2] and observational studies [3, 4] links UPF consumption with positive energy balance and increased risk of obesity, but the role of alcohol is unresolved. Despite its high energy density (29 kJ/g) and experimental evidence that alcohol calories are poorly compensated by reductions in macronutrient energy [2, 3, 4, 5], results from both observational and experimental studies are mixed, showing increased, decreased, or no effect on energy intake [5, 6]. This suggests that the effects of alcohol on energy balance may be more complex than appreciated, and contingent on one or more mechanistic and/or ecological moderators that influence the strength and/or sign of the relationship [7, 8]. Considering the established influence of UPFs on energy balance, and that the consumption of UPF may be socio‐ecologically associated with alcohol consumption [7], we developed and tested a mechanistic‐ecological model to examine the hypothesis that dietary pattern is a key moderator that could explain the variable association between alcohol and energy balance.

Mechanistic‐ecological models integrate experimental and observational evidence to understand complex phenomena, using controlled experiments to generate causally grounded predictions and observational data to test them in relevant ecological settings. They thus combine the strength of experiments for establishing causality with the ecological relevance of observation [8]. In this framework, the role of observational data is explicitly to test the ecological validity of a causally grounded model, not to establish causality per se. Mechanistic‐ecological models have been used in the epidemiology of human [9, 10] and animal [11] disease, in agriculture [12], and in the ecological sciences [13]. However, despite their promise for strengthening nutrition policy [14], mechanistic‐ecological models are scarcely used in human nutrition.

A challenge to understanding the role of alcohol in energy balance and weight regulation is that alcohol could interact in complex ways with dietary macronutrient mixtures [15], which themselves influence food and energy intake [16, 17]. A recent systematic review reported substantial variation in the results of 18 experimental and 12 observational studies examining how alcohol influences dietary selection and concluded overall that the evidence suggests complex dose‐dependent interactions between alcohol and macronutrient intake [17]. The Geometric Framework for Nutrition (GFN), a multidimensional framework [18, 19] developed for modeling nutrition as interactions of consumer behavior and physiology with dietary mixtures [20], provides a tool for examining the relationships between alcohol intake, foods, dietary macronutrient compositions, and energy intake.

The GFN has been used in randomized clinical trials to examine how dietary mixtures influence macronutrient and energy intakes [21, 22]. A consistent finding is that humans, in common with several other species of primates [23], regulate the intake of protein more tightly than other macronutrients or micronutrients [19, 24], and consequently food intake increases to compensate for dietary protein dilution, a phenomenon called protein leverage [19, 22, 25]. When the diluent of protein is macronutrient energy (carbohydrate and fat), protein leverage results in increased energy consumption and potentially positive energy balance and weight gain [22]. Protein leverages the intake of both carbohydrate and fat, but is expected to do so more strongly for fat owing to its high energy density [16]. Several population studies of free‐living humans have, likewise, found that protein intake is maintained more constant than fat or carbohydrate, and consequently energy intake varies inversely with percentage dietary energy contributed by protein, as predicted if protein leverage is operational in ecological settings [3, 26, 27, 28, 29, 30].

Experimental evidence from both rodents and humans links alcohol consumption to protein leverage via the endocrine hormone fibroblast growth factor 21 (FGF21). Circulating FGF21 is elevated under low protein intakes and acts via the central nervous system to increase protein intake [15, 31, 32, 33, 34, 35, 36, 37, 38, 39, 40, 41]. It does so by increasing preference for umami‐flavored (savory) foods (the “aperitif effect”) and decreasing preference for sweet foods, and—when subjects are restricted to low‐protein diets—through a compensatory increase in food intake (protein leverage) [31, 42]. Alcohol intake is also a strong acute inducer of circulating FGF21, which remains elevated for several hours after consumption, or for longer periods under subchronic exposure [31, 33, 43, 44, 45, 46, 47, 48, 49, 50, 51, 52, 53]. Alcohol consumption can thus potentially mimic the effects of low‐protein status on appetite and energy balance, increasing protein appetite and stimulating umami‐seeking, thereby influencing patterns of food and macronutrient intakes [17, 54, 55, 56].

In ecological settings, however, the effects of FGF21‐induced umami‐seeking and increased protein appetite will vary with dietary exposures. In minimally processed dietary patterns, umami flavors are correlated with high protein content and thus provide a reliable sensory predictor of protein [54, 55, 56]. In modern industrialized food environments, in contrast, the correlation between umami flavor and protein is subverted by the widespread use of additives such as artificial umami flavorings and salt that mimic protein in fat‐ and carbohydrate‐rich savory UPFs [57], and by consumption of unprocessed high‐fat meats, both of which effectively act as “protein decoys” [42]. Alcohol consumption could thus have contrasting effects on energy intake depending on the extent to which it is consumed in association with minimally processed vs. ultra‐processed/high‐fat meat dietary patterns. We hypothesized that the inconsistent results of previous studies reflect this FGF21‐mediated interaction, in which dietary pattern modulates the effect of alcohol on macronutrient energy intake.

**FIGURE 1:**
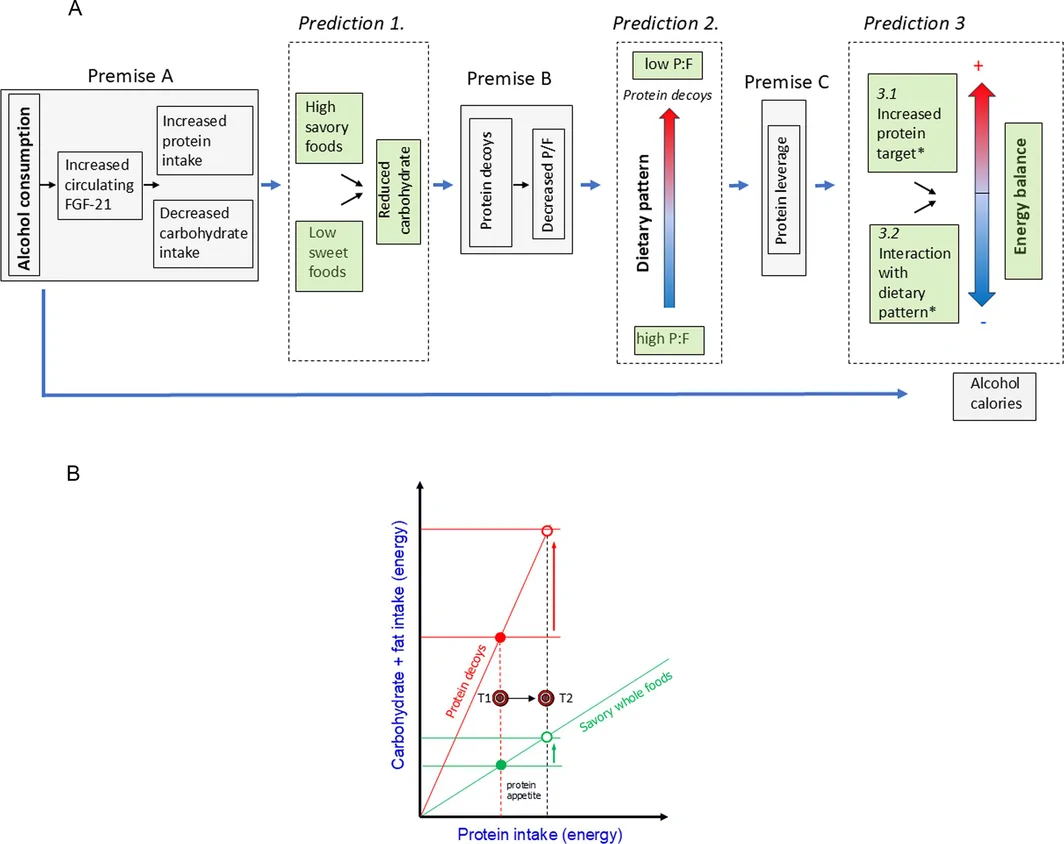
Mechanistic‐ecological model of interactions between alcohol intake, macronutrient regulation and dietary pattern. (a) Blue arrows indicate links between the premises (previously established mechanisms, see Introduction) and associated predictions to be tested in ecological settings (see Results). From left to right, alcohol consumption induces elevated circulating FGF21, which stimulates protein intake and decreases carbohydrate intake (Premise A), giving rise to Prediction 1: that the intake of savory‐flavored foods would be high and sweet‐flavored foods low in alcohol consumers, with an associated decrease in dietary carbohydrate. On dietary patterns dominated by whole foods, where umami flavour typically predicts protein, this will translate to increased dietary protein:fat (P:F); in contrast, on dietary patterns rich in “protein decoys” (umami‐flavored ultraprocessed savory snacks and high‐fat meats), dietary P:F will be reduced when consuming alcohol (Premise B). Prediction 2 is therefore that dietary P:F would vary negatively with the relative contribution of protein decoys in the diet. Because protein intake is regulated to a target level while nonprotein energy intake is allowed to vary more passively with dietary composition (protein leverage, Premise C), alcohol‐induced elevation of FGF21 is predicted to produce a spectrum of energy intakes that span positive and negative energy balance depending on dietary P:F ratio. Prediction 3 is based on two mechanisms (further explained in Figure 1b). First, alcohol‐stimulated FGF21 should result in an increase in the protein intake target, and an associated increase in total food and energy intake (Prediction 3.1). Second, the increase in energy intake associated with a given increase in protein intake will be disproportionately larger on low P:F diets (e.g., those rich in protein decoys), such that the alcohol‐associated increase in total energy intake is predicted to vary along a spectrum according to the P:F ratio of the dietary pattern (Prediction 3.2). (b) Schematic showing the anticipated effect of an alcohol‐induced increase in the protein intake target. T1 represents the baseline intake target, which is elevated along the protein axis to T2 upon alcohol consumption. The red radial represents a low protein:(fat + carbohydrate) savory food (“protein decoy”), and the green radial represents a savory whole food (typically high in protein). Because in humans, protein intake is regulated more tightly to a target level than carbohydrate and fat (protein prioritization; the vertical dashed lines), the intake of carbohydrate and fat is expected to be inversely proportional to dietary protein:(carbohydrate + fat) ratio (solid green and red symbols) (protein leverage, Premise C). With an alcohol‐induced increase in protein intake from T1 to T2, carbohydrate and fat intake are also increased (Prediction 3.1), but to a greater extent on low‐protein than high‐protein diets (red and green arrow, respectively).

We used the GFN to integrate evidence from controlled preclinical studies and clinical trials in a mechanistic‐ecological model predicting how alcohol‐induced FGF21 elevation could interact with food selection and macronutrient regulation to influence energy intake, depending on the extent to which alcohol is consumed with protein decoys. Our model (Figure 1) predicts that alcohol intake will be associated with consumption of umami‐flavored foods and reduced consumption of sweet‐flavored foods, but that its effect on energy intake and energy balance will vary depending on the proportion of protein decoys vs. minimally processed protein‐rich foods with which it is consumed. We tested these predictions in an ecological setting by using Nutritional Geometry to analyze cross‐sectional data from a large free‐living human population, the Australian National Nutrition and Physical Activity Survey (NNPAS). The results bear close resemblance to the detailed predictions of the model, potentially reconciling contradictory findings in the literature and suggesting new avenues for experimental and epidemiological research that could inform novel public health interventions.

## 2 Methods

### 2.1 Model and Predictions

Our model combines published mechanistic evidence from experimental research on humans and animal model systems (see Introduction) to examine how inconsistent results in research on the relationship between alcohol consumption and energy intake might be reconciled (Figure 1A,B). The evidence was interrelated within the Nutritional Geometry Framework (NGF) to determine how alcohol consumption might interact with macronutrient mixtures to influence diet selection and consumption across varying dietary patterns. In this approach, the experimentally established mechanisms provide the causal foundations of the model, while the observational data (see Population Study Design) serve explicitly to test its predictions in an ecological setting—assessing external validity rather than establishing causality. The model comprises three premises (A, B, C), each representing an experimentally established mechanism described in the Introduction, from which three predictions are derived (Figure 1A). For clarity, we present each premise together with its associated prediction and results in the Results section below.

### 2.2 Population Study Design

The Australian NNPAS is a cross‐sectional, stratified multistage area sample of private dwellings conducted by the government organization, the Australian Bureau of Statistics (ABS) [58].

#### 2.2.1 Settings

The NNPAS was conducted as part of the 2011–2012 Australian Health Survey, to acquire comprehensive national benchmark data on nutrition and physical activity. Information was collected during May 29, 2011 and June 9, 2012 with interviews conducted Monday to Sunday across the year. Response rates for each day were as follows: Sunday 18.3%, Monday 18.8%, Tuesday 18.1%, Wednesday 16.5%, Thursday 13.9%, Friday 11.1%, and Saturday 3.4%. The NNPAS interviews were held with 9500 private dwellings selected throughout Australia except for very remote areas. The sample was constructed such that each person in Australia had an equal chance of being selected. Data were collected for one adult and one child aged 2–17 years from each of the households selected. A face‐to‐face interview was conducted for each adult member in a computer‐assisted personal interview (CAPI) by trained ABS interviewers. All participants were asked to attend a second 24‐h recall over a computer‐assisted telephone interview (CATI) at least 8 days later.

#### 2.2.2 Participants

Eligible participants were self‐identified and included usual residents of private dwellings in rural, regional, and urban areas of Australia, comprising 97% of the Australian population. Overseas visitors residing in Australia for 12 months or more were considered within scope. The survey excluded residents of nonprivate dwellings (e.g., boarding schools, hospitals, and hotels), which was approximately 2% of the population, and households of defense forces, diplomats, and those living in very remote areas of Australia. These exclusions are unlikely to affect national estimates.

A total of 12,153 participants reported data in the 2011–2012 NNPAS. After excluding children, there were 9337 adults aged 19 years and over. Of these, 3082 (35.1%) reported alcohol consumption on the day of survey. A second day of nutrition recall was collected for a total of 6113 adults (64.8%). Participant characteristics are described in Table S1.

#### 2.2.3 Variables

Outcomes of interest (including total daily energy intakes, alcohol intakes, and amounts eaten of different categories of foods) were expressed as absolute values in kilojoules or grams, and mapped against dietary macronutrients expressed as the proportion of energy from carbohydrate, fat, and protein (i.e., macronutrient energy 100% = protein (%) + fat (%) + carbohydrate (%)).

Our primary aim was to use the widely known NOVA food classification system, in which the not recommended category, UPFs (NOVA 4), have previously been identified as contributing disproportionately to excess energy intake and positive energy balance. However, a subset within the NOVA 1 unprocessed foods category, high‐fat minimally or unprocessed meats, has the same combination of intrinsic properties implicated in the interaction of UPF with alcohol (umami flavor paired with low P:F), and on this basis has previously been classified together with savory UPF as “protein decoys” [42]. Since the Australian Dietary Guidelines (ADG) food classification system recommends against consumption of both high‐fat meats and highly processed (“discretionary”) foods, we therefore also analyzed the data across recommended and not recommended foods under the ADG for comparison with NOVA. Previous comparisons of ADG and NOVA showed a high degree of congruence (~75% of foods are classified similarly) between UPFs and “discretionary” foods in Australia [59]. Our analysis showed no qualitative difference in outcome according to which system was used, but the effects of alcohol on energy intake were notably more pronounced when using the ADG (as demonstrated in Results).

Mixed dishes were disaggregated into their component foods. Foods were classified using both the ADG system of recommended “five food groups” vs. “not recommended” (high‐fat meats and discretionary foods) [60], and according to the NOVA classification system by the degree of processing into “minimally processed”, “culinary ingredients”, “processed foods”, and “ultra‐processed foods” [61]. The ADG‐recommended “five food groups” include: fruit; vegetables and legumes; milk, yoghurt and cheese, and their alternatives; grains and cereals; and lean meat, poultry, fish, seafoods, nuts, seeds, tofu, eggs, and beans and legumes. “Discretionary foods” are classified to include foods high in saturated fats, added sugars, added salt and/or alcohol, e.g., sausages, processed meats, commercial burgers, potato chips, cakes, biscuits, confectionary, chocolate, sausage rolls, meat pies, butter, ice cream, cream, sugar‐sweetened beverages, and alcoholic beverages. Not recommended high‐fat meats are considered to be those with greater than 10% fat by wet weight. UPFs in the NOVA classification are defined as foods that are often highly concentrated in culinary ingredients (fats and sugars) and include industrial ingredients such as colors, dyes, flavor enhancers, flavorings, emulsifiers, or food extracts such as hydrolyzed proteins, hydrogenated oils, high fructose, and maltodextrin. Further details of the classification are described in detail elsewhere [61].

To test our predictions, we further subdivided relevant categories within each system according to their principal flavor characteristics (savory or sweet) because flavor is the organoleptic property most directly implicated in our model—specifically, umami, the principal gustatory signal for protein appetite stimulated by FGF21 elevation, and sweetness, the property suppressed by FGF21 elevation. Umami‐flavored foods were classified into three groups: Group 1—included whole foods with less than 10% fat by wet weight, e.g., lean beef, pork, lamb, poultry, fish, seafood, eggs, tofu, nuts, and seeds; Group 2—were all high‐fat unprocessed meats with ≥10% wt. fat, e.g., higher‐fat meat cuts such as duck consumed with skin and fat, pork belly, and those described as untrimmed and semi‐trimmed meat, poultry and fish; Group 3—included savory “discretionary” foods, including savory crackers, savory pastries, meat pies, sausage rolls, savory sauces, commercial dips, potato fries, and other savory snack foods including potato crisps, extruded snacks, corn chips, and all processed meats (salami, ham, bacon, processed poultry and deli meats, and sausages). Additionally, amounts of savory UPFs were calculated, which included all savory foods classified as UPF within the NOVA classification system, including instant noodles, savory biscuits, savory pastries, frozen fish and fish products, sausages, ham, salami and other ultra‐processed meats and chicken, chicken nuggets, packet and canned soups and sauces, commercial dips, frozen and commercial potato products and fries, potato and corn crisps and other extruded snack foods, and commercial stocks.

Sweet foods included all sweet highly processed foods as described by the ABS (e.g., cakes, sweet biscuits, sweet pastry, sugar‐sweetened beverages, table sugar, ice cream and ice confection, jams, confectionary, chocolates, and breakfast cereals high in added sugar).

#### 2.2.4 Data Sources—Measurements

Twenty‐four‐hour recall data form the main source of dietary data. The questionnaire collected detailed information on all food and beverages consumed within a 24‐h period from midnight to midnight on the day prior to the interview. The method for the 24‐h recall was the Automated Multiple‐Pass Method (AMPM) developed by the Agricultural Research Service of the United States Department of Agriculture (USDA). The ABS and Food Standards Australia New Zealand (FSANZ) modified the AMPM instrument to reflect the Australian food supply. The AMPM is an automated questionnaire. The tool contains more than 10,000 individual foods and food combinations that capture the Australian food supply.

Amounts of food and beverages consumed were estimated with the assistance of the Food Model Booklet. The booklet contained actual size photographs and drawings of typical container and serve sizes sourced from Australia.

**FIGURE 2:**
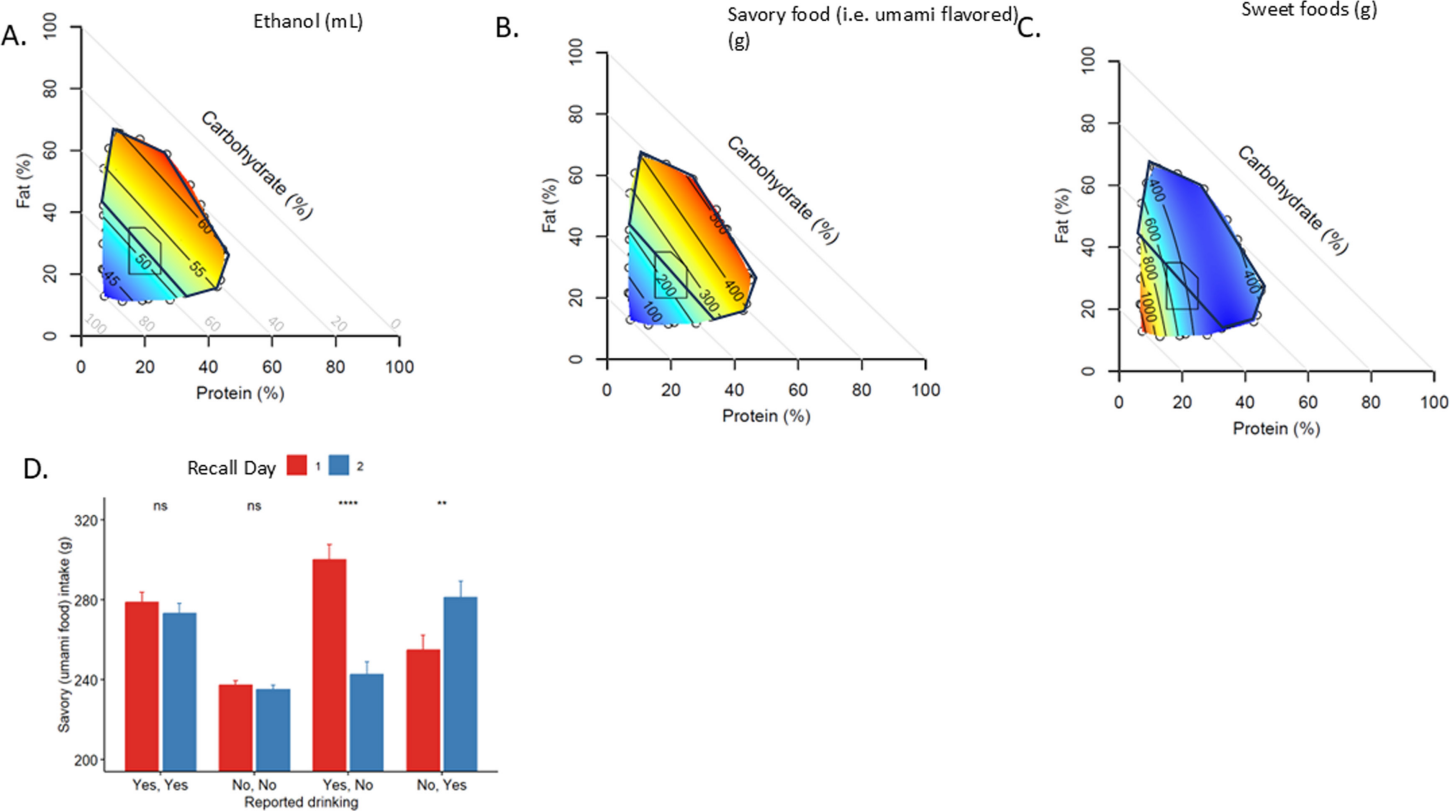
Right‐angled mixture triangle surface plots (A–C) showing associations between dietary macronutrient mixtures and reported consumption of pure alcohol (ethanol) (A), umami‐flavored foods (B), and sweet foods (C) for adults who reported alcohol consumption on the primary survey day (*n* = 3139). Highest values on the surface plots are shown in red and lowest values in deep blue. For each diet composition point (beneath the response surface), protein (*x* axis), fat (*y* axis), and carbohydrate (increasing across the gray negative diagonals towards the origin) sum to 100%. The fine‐lined polygon represents the Australian/New Zealand Acceptable Macronutrient Distribution Range (AMDR). The bold‐lined polygon encloses the region of alcohol intakes above the single occasion risk guidelines of 50.8 mL (40 g ethanol; four standard drinks). Consistent with Prediction 1, higher ethanol intake was associated with increasing percent dietary protein and fat and decreasing carbohydrate (A). Savory food intake (B) closely mirrored the distribution of alcohol intake (A), whereas sweet food intake (C) showed the opposite relationship. Data are adjusted for socio‐economic position, age, and sex. Panel D shows savory (i.e., umami‐flavored) food intake on the first and second 24‐h recall for all participants that were surveyed on two separate days (*n* = 6113) grouped by survey day that drinking was reported. Significant differences determined with paired *t*‐tests are indicated by asterisks: **p < 0.001, ***p < 0.0001, ns = not significant. Savory food intake was significantly higher on drinking days than nondrinking days, suggesting that the association reflects within‐person dietary shifts toward umami on days alcohol is consumed, rather than a between‐person co‐occurrence of high alcohol and high umami preference.

The second interview was ideally conducted on a different day of the week from the first interview. However, this was not always possible. A black and white version of the food model booklet was given to participants to use in the second interview. As only 64.8% of participants reported data on the second interview, only the first day of the data was utilized in the main analysis. However, in a separate within‐subjects analysis, diets were compared between the first and second survey days for the subset of respondents who completed both interviews (Figure 2D).

Nutrient composition was estimated with the AUSNUT 2011–13 food nutrient database. The database contains 53 nutrient values for 5740 foods and beverages that were consumed during the 2011–12 NNPAS. The nutrient composition of the foods and beverages in AUSNUT reflect the Australian food supply and food preparation methods during 2011 to 2012.

FGF21 was not measured in the survey and therefore FGF21 cannot be directly assessed in this analysis; rather, it was inferred from randomized control trials demonstrating an association between alcohol consumption and elevated circulating FGF21 (see Introduction).

#### 2.2.5 Statistical Methods

Macronutrient intakes were normally distributed. Means and linear regressions were conducted in SAS (PROC SURVEYMEANS and PROC SURVEYREG) and used to test the relationship between protein, alcohol intake, and food intakes for alcohol consumers and nonconsumers. Models were adjusted for age, sex, and socio‐economic index for area (SEIFA). Power regression (SPSS IBM SPSS Statistics v. 25) was used to test protein leverage [19, 26, 28], where if protein intake (*P*) stays constant and nonprotein energy varies exponentially with changing dietary proportion of energy from protein (*p*), the exponent (*L*) takes a value of −1, in the equation *Pp* ^*L*^. Complete protein leverage (*L* = −1) is indicated if absolute protein intake is maintained constant while total energy intake varies inversely with the dietary proportion of energy from protein. Protein leverage is partial if *L* > −1 but <0, in which case energy intake varies inversely with dietary protein density, but protein intake is not maintained constant, increasing with protein density. There is no evidence for protein leverage if energy intake does not vary (*L* = 0) or varies positively (*L* > 0) with dietary protein density.

Macronutrient mixtures were visualized as right‐angled mixture triangles with surface plots for energy, food intake, and alcohol, adjusted for age, sex, and socio‐economic position measured with quintiles determined by the ABS SEIFA [20, 57]. Scheffe's polynomials, or mixture models, were fitted to the data for total energy intake, alcohol, and food intakes for alcohol consumers (main results) only and for all participants (Supplementary Tables 2–15) using R packages, mixep, and sp. Models with the smallest Akaike Information Criterion were used to estimate the response surface. Detailed explanations of the models are published elsewhere [20, 62, 63]. Participants with macronutrient ratios within 3 standard deviations of the log of the mean were included for analysis to remove the effect of macronutrient ratios outside the boundaries of usual intakes. Survey weights provided by the ABS were used to calculate means and proportions of respondents of different demographics to provide point estimates for the Australian population, accounting for nonresponse bias and the complex survey design.

## 3 Results

Results are organized to follow the logical sequence of our model (Figure 1a), presenting each experimentally grounded premise alongside its prediction and the associated observational evidence.

### 3.1 Premise A and Prediction 1

#### 3.1.1 Premise A

Alcohol consumption increases circulating FGF21, which signals low‐protein status and acts via the central nervous system to increase protein intake and decrease carbohydrate intake (see Introduction).

#### 3.1.2 Prediction 1

Alcohol consumption is associated with increased selection of umami‐flavored food, decreased sweet preference, and decreased proportional carbohydrate intake.

Alcohol intake plotted over the percentage energy from protein, fat, and carbohydrate for consumers of alcohol is shown in Figure 2A, adjusted for age, sex, and SEIFA. The large polygon represents the region in which reported alcohol intake exceeds the recommended intake in a single occasion of 50.8 mL (40 g) of ethanol (four standard drinks). Consistent with Prediction 1, high ethanol intakes were strongly associated with decreasing proportion of energy contributed by carbohydrate (Figure 2A), high intake of savory foods (Figure 2B), and low intakes of sweet foods (Figure 2C) on the survey day. The adjusted mean (SE) grams consumed of umami‐flavored foods were significantly greater for alcohol consumers compared to nonconsumers at 276.1 g (4.7) vs. 235.6 g (3.1) (*p* < 0.001), respectively. Daily sweet food intake was lower at 294.2 g (10.3) for alcohol consumers compared to 345.0 g (7.5) (*p* < 0.001) for nonconsumers. Including nonconsumers as 0 g alcohol intake, umami‐flavored food increased by 7.1 g (1.0) for each standard drink (*P* = <0.0001) and sweet foods decreased by 10.7 g (3.4) per standard drink (*p* = 0.003). Among subjects who were surveyed on 2 days, savory food consumption was higher on days they reported alcohol vs. not (Figure 2D). This analysis, which controls for all lifestyle factors because each participant acts as their own control, suggests that alcohol consumption is associated with dietary shifts toward increased umami rather than high alcohol and umami preference co‐occurring in subsets of the population.

### 3.2 Premise B and Prediction 2

#### 3.2.1 Premise B

Protein decoys (i.e., savory UPFs and high‐fat meats) are typically lower in protein and lower in protein:fat ratio compared to most savory whole foods (see Introduction).

#### 3.2.2 Prediction 2

Dietary protein:fat ratio is inversely related to the contribution of protein decoys in the diet.

**FIGURE 3:**
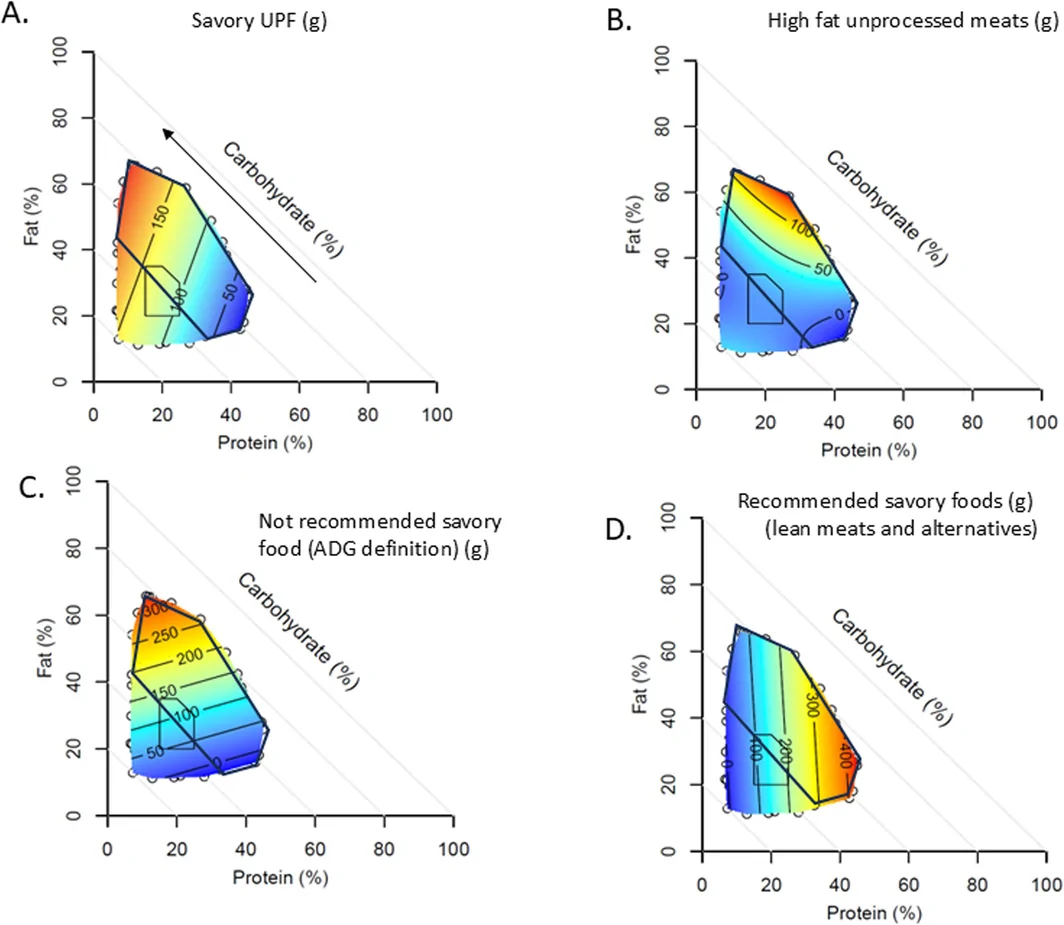
Right‐angled mixture triangle (RMT) surface plots for alcohol consumers showing relationships between three‐dimensional dietary macronutrient composition (protein, fat, carbohydrate; see Figure 2 for an explanation of the RMT axes) and reported consumption of categories of savory foods. The diagonal arrow in the first panel highlights the direction of decreasing protein:fat ratio. (A) Savory UPFs; (B) High‐fat unprocessed meats (e.g., untrimmed meats such as pork belly and duck, and other red meat, poultry, seafood, and fish >10 g/100 g fat); (C) “Not recommended” savory foods (i.e., savory “discretionary” foods and high‐fat unprocessed meats, combined = protein decoys); (D) “Recommended” savory foods (lean red meat, poultry, fish, seafood, nuts, seeds, and legumes). The region enclosed by the black hexagon represents alcohol intakes above the single occasion risk guidelines of 50.8 mL (40 g ethanol; four standard drinks). Data adjusted for socio‐economic position, age, and sex. Savory UPF (A) and high‐fat unprocessed meats (B) were both concentrated on low‐protein:fat diets, with their combined contribution (C) exceeding either alone—a pattern better captured by the ADG classification than by NOVA, which does not classify unprocessed meats as a category to be avoided.

Consistent with Prediction 2, the dietary ratio of protein:fat decreased with increasing contribution of savory UPF in the diet (Figure 3A). As anticipated, this relationship was not unique to UPF but shared with high‐fat unprocessed meats (Figure 3B). The gradient was more pronounced when savory “discretionary” foods—which largely but not entirely overlap with savory UPF—were combined with high‐fat unprocessed meats in the ADG “not recommended” category (Figure 3C). The opposite relationship, of increasing protein:fat, was observed among savory foods in the recommended category under the ADG (Figure 3D). These results suggest that the ADG classification, by flagging both “discretionary” foods and high‐fat unprocessed meats as “not recommended”, better captures the combined dietary drivers of protein dilution than the NOVA system, which classifies high‐fat unprocessed meats as NOVA 1 (minimally and unprocessed foods) and therefore outside of the “not recommended” category.

### 3.3 Premise C and Prediction 3

#### 3.3.1 Premise C

**FIGURE 4:**
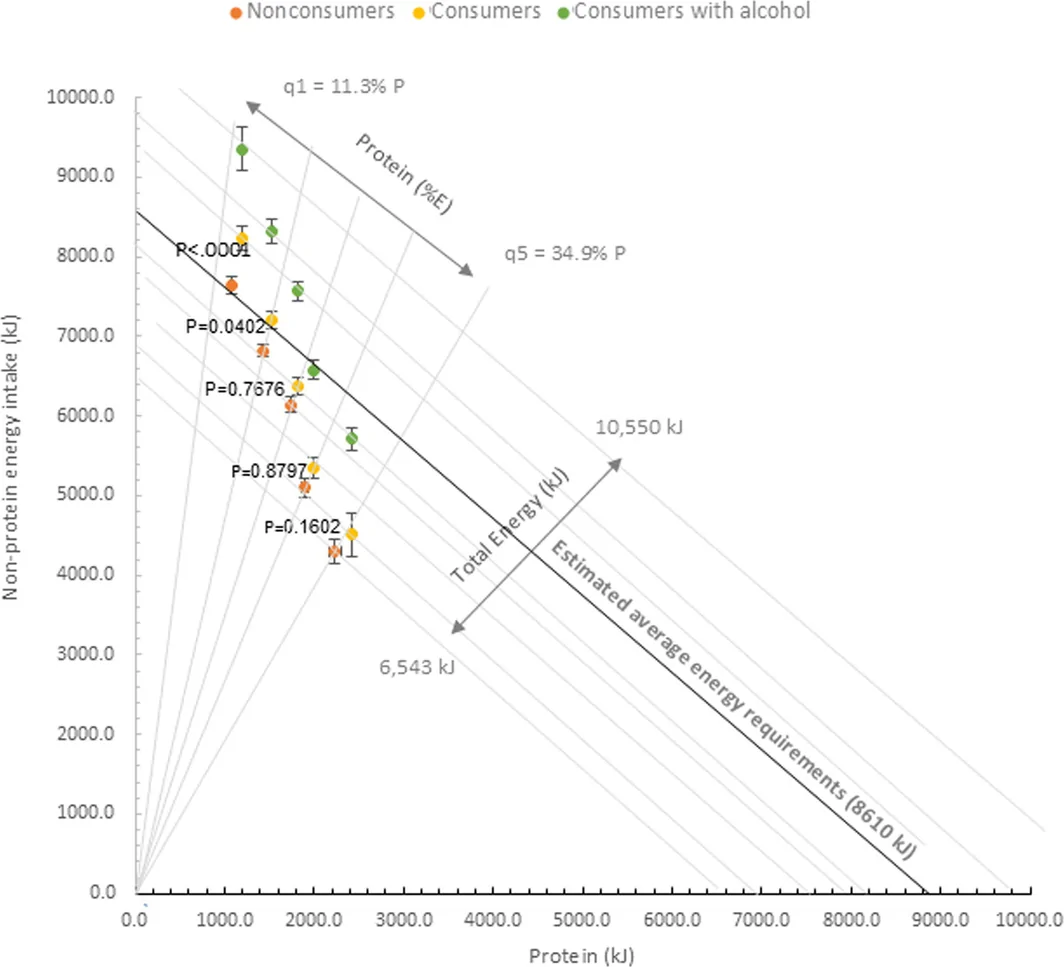
Relationship between protein and nonprotein macronutrient energy (i.e., fat + carbohydrate) intakes partitioned by quintiles of dietary proportion of protein energy for alcohol consumers (orange) and nonconsumers (yellow). Both groups of respondents maintained absolute protein intake relatively tightly (i.e., the array of intakes fall along a line that has a steeper slope than if total energy was prioritized), with nonprotein energy intake varying more widely with variation in dietary macronutrient ratios—indicative of protein leverage (Premise C). Total energy intake increased from 6543 kJ for quintile 5 (highest % protein labelled q5) to 9550 kJ for quintile 1 (lowest % protein, q1). Consistent with Prediction 3.1, protein intakes and associated total energy intakes were higher for alcohol consumers than nonconsumers across all quintiles. Consistent with Prediction 3.2, the difference in energy intakes between consumers and nonconsumers was disproportionately greater on low‐protein diets (top left of figure). The black diagonal line in the graph represents the estimated average daily energy requirement for the survey participants in the normal weight range, assuming energy at equilibrium and physical activity level of 1.4. All data are adjusted for age, sex, and socio‐economic position.

Food intake varies negatively with dietary protein density, as expected under protein leverage (see Introduction). Alcohol‐induced elevation of FGF21 is predicted to interact with protein leverage and dietary pattern to influence macronutrient energy intake through two related mechanisms, giving rise to two subpredictions (as illustrated in Figure 1). Both subpredictions are tested together in Figure 4, which plots absolute protein against nonprotein energy intake across quintiles of dietary protein for alcohol consumers and nonconsumers.

#### 3.3.2 Prediction 3.1

FGF21‐stimulated protein appetite effectively increases the protein intake target (Figure 1B), requiring greater total energy consumption at any given dietary protein:nonprotein energy ratio. Alcohol consumers are therefore expected to have higher intakes of both protein and nonprotein macronutrient energy than nonconsumers on nutritionally equivalent diets.

#### 3.3.3 Prediction 3.2

This effect will be disproportionately pronounced on ultra‐processed dietary patterns, because the lower protein density of protein decoys means that a greater total food intake is required to reach the elevated protein target, amplifying protein leverage and its associated effects on energy intake (Figure 1B).

In both alcohol consumers and nonconsumers, absolute protein intake (kJ) remained more constant than nonprotein energy, and consequently energy intake increased across quintiles of decreasing dietary percentage energy from protein, indicative of protein leverage (Premise C; alcohol consumers: *L* = −0.23, *p* < 0.001; nonconsumers *L* = −0.27, *p* < 0.0001). In accordance with Prediction 3.1, protein intakes were significantly higher for consumers of alcohol than nonconsumers (mean = 91.5 g (0.8) vs. 86.2 g (0.7), respectively (*P* = <0.001)), as were macronutrient energy intakes (mean = 8428.4 kJ (63.2) vs. 7992.9 (72.0) kJ, respectively (*p* < 0.0001)). Consistent with Prediction 3.2, the difference in macronutrient energy intakes between alcohol consumers and nonconsumers increased with decreasing dietary percentage of energy contributed by protein (*p* = 0.0144 for the interactive effect of alcohol consumption × dietary quintile % energy from protein). Together, these results support both subpredictions: alcohol consumers regulate protein intake at a higher protein target, and this effect is amplified on protein‐dilute diets, as expected if FGF21‐stimulated protein appetite interacts with protein leverage.

**FIGURE 5:**
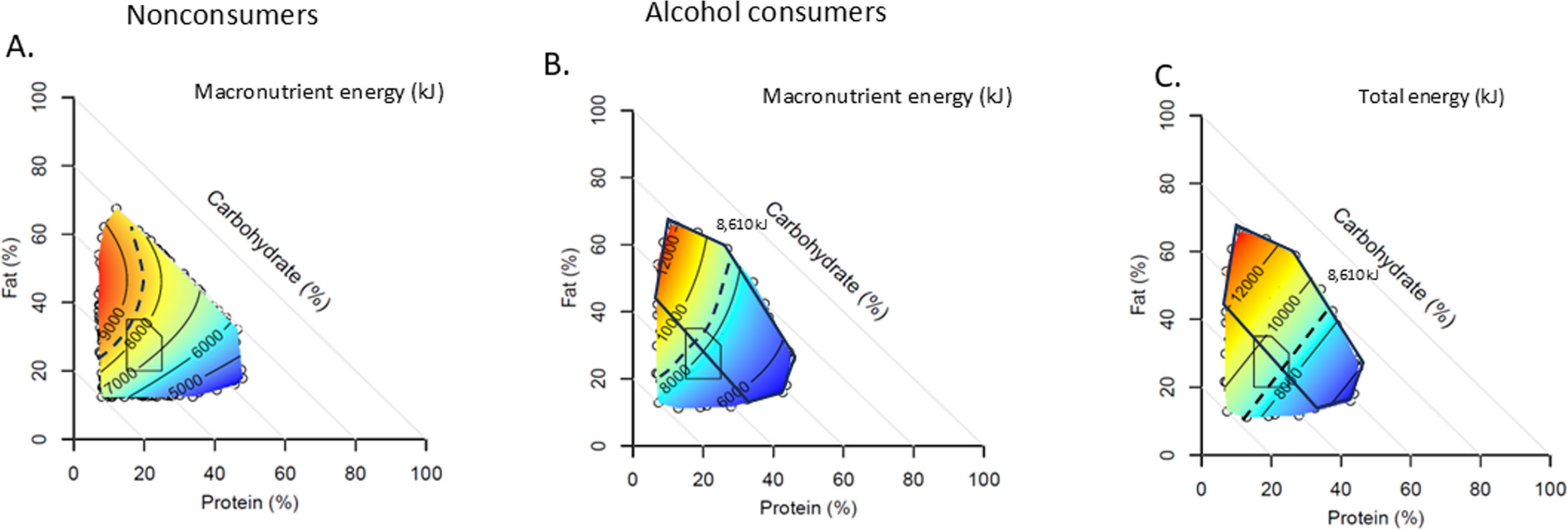
Extending the test of Prediction 3 from Figure 4 into the full three‐dimensional compositional space, macronutrient energy intakes are mapped onto the dietary macronutrient landscape for nonconsumers (A) and consumers (B) of alcohol, and total (macronutrient + ethanol) energy intake for alcohol consumers (C) (see Figure 2 for axis explanation). The figure shows that whereas energy intake in nonconsumers peaked across a wide range of fat:carbohydrate ratios on low‐protein diets (vertical red strip, A), in consumers it was concentrated specifically on low‐protein:fat diets (B, C)—the same dietary region occupied by protein decoys (Figure 3)—enabling the interaction between alcohol consumption and protein leverage to be examined in relation to specific food categories (Figure 6). The dashed contour at 8610 kJ marks the recommended daily energy intake. The surfaces show that the interaction of alcohol consumption with dietary P:F ratio generates the expected spectrum of energy balance outcomes, with alcohol consumer intakes spanning from below this threshold on high P:F diets to substantially above it on low P:F diets—a range that widens further when alcohol energy is included (C). Sample sizes: nonconsumers *n* = 6062; alcohol consumers *n* = 3085. All data adjusted for age, sex, and socio‐economic position.

Extending the test of Prediction 3 into three‐dimensional composition space, Figure 5 shows macronutrient energy intakes and total energy intakes (macronutrient + ethanol) mapped onto dietary macronutrient space for nonconsumers and consumers of alcohol. The increase in macronutrient energy intake with decreasing dietary % protein can be seen both for nonconsumers (Figure 5A) and consumers of alcohol (Figure 5B), but the topography of the surfaces for the two groups differed in two key respects. First, for nonconsumers, energy intakes peaked on low‐protein diets spanning a wide range of fat:carbohydrate ratios (vertical red strip), whereas for alcohol consumers, macronutrient energy intakes peaked specifically on diets with low‐protein:fat ratios (Figure 5B,C). Second, the macronutrient energy intake peak was higher for consumers (c. 11,000–12,000 kJ; Figure 5B) than nonconsumers (c. 9000–10,000 kJ; Figure 5A), with consumer intakes in the low‐protein:fat region exceeding the recommended daily energy intake of 8610 kJ/day (shown as a contour in Figure 5), a gradient that was further accentuated when including energy consumed as alcohol (c. 12,000–13,000 kJ; Figure 5C). Together, these surfaces show that the interaction of alcohol consumption with dietary P:F ratio generates the predicted spectrum of intakes, from below the daily recommendation (negative energy balance) to above (positive energy balance), and this difference widens further when alcohol energy is included (Figure 5C).

**FIGURE 6:**
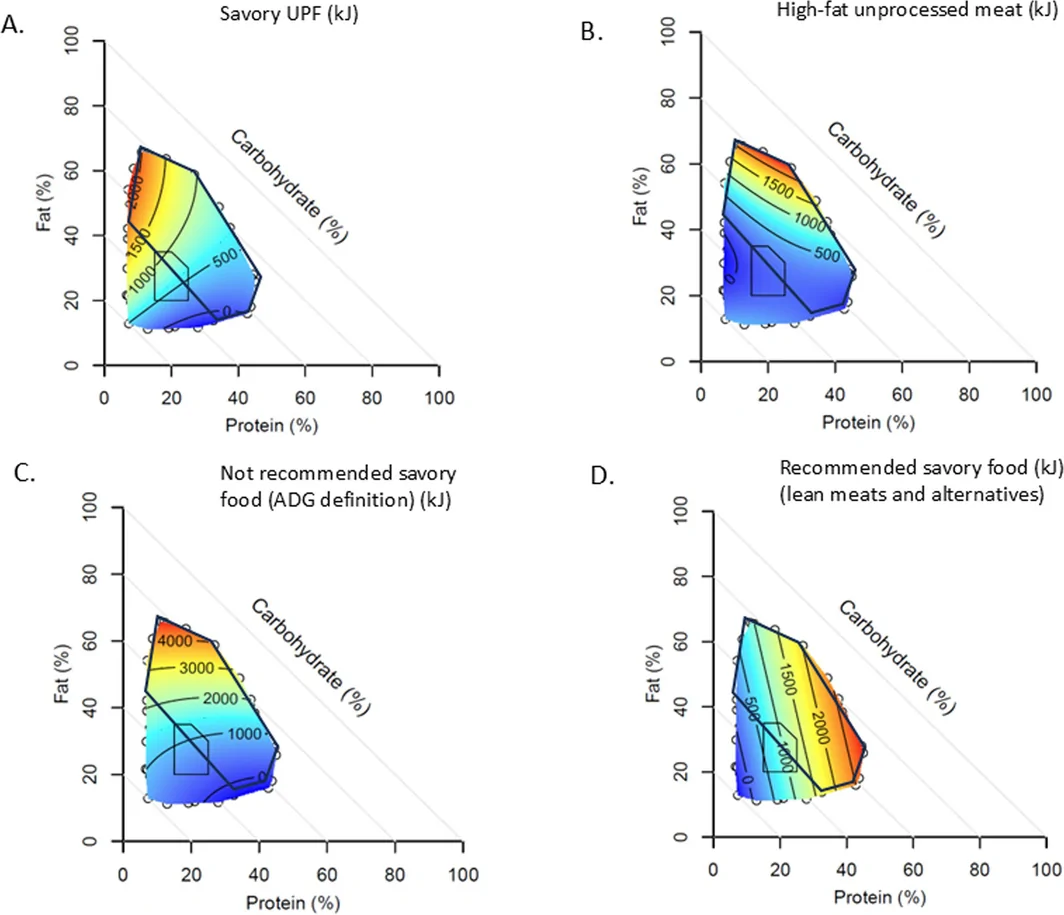
Right‐angled mixture triangle surface plots (see Figure 2 for axis explanation) showing associations for alcohol consumers between macronutrient distributions and daily dietary energy contributed by different categories of foods as defined in Figure 3: (A) savory UPF; (B) high‐fat unprocessed meats; (C) ADG “not recommended” savory foods (discretionary foods and high‐fat unprocessed meats combined); (D) ADG “recommended” savory foods. Consistent with Prediction 3, energy contributions from savory UPF (A) and high‐fat unprocessed meats (B) were concentrated on low‐protein:fat diets, with their combined contribution in the ADG “not recommended” category (C) exceeding either alone. This reveals these food categories as the primary contributors to elevated energy intake in alcohol consumers on low‐protein:fat diets. “Recommended” savory foods showed the opposite pattern (D), with energy contributions increasing toward high‐protein:fat (low energy intake) diets. Data adjusted for age, sex, and socio‐economic position.

Expressing the protein leverage × ethanol interaction in a three‐dimensional compositional space allows us to examine the food categories underlying it. The low‐protein:fat dietary region where consumer energy intakes peaked corresponds to diets rich in savory UPF (Figure 3A) and high‐fat unprocessed meats (Figure 3B), and the contributions of these and other savory food categories to macronutrient energy intakes across this landscape are shown in Figure 6. Energy contributed by savory UPF increased with decreasing protein:fat ratio, to a maximum of 2000 kJ (top left, Figure 6A), consistent with these foods being primary protein decoys operating in low‐protein:fat dietary regions. A similar pattern was shown for high‐fat unprocessed meats, also reaching a maximum of 2000 kJ in the low‐protein:fat region (Figure 6B), confirming that, in Australia, high‐fat meats constitute a second route to low‐protein:fat diets among alcohol consumers, as anticipated under Premise B. The savory foods “not recommended” under ADG, which comprise both “discretionary” foods and high‐fat unprocessed meat, thus contributed twice the dietary energy for alcohol consumers on low‐protein:fat diets than did UPF alone (Figure 6C). “Recommended” savory foods showed the opposite pattern, reaching a maximum contribution of 2500 kJ on diets with high‐protein:fat (Figure 6D).

Overall, these results accord with Prediction 3, that the diets of high alcohol consumers would span macronutrient and total energy intakes that vary substantially in relation to the recommended daily intakes (8610 kJ/day), depending on the dietary pattern.

## 4 Discussion

There is strong evidence linking UPFs to energy overconsumption, obesity, and associated disease [64, 65, 66]. In contrast, there is much uncertainty over the contribution of alcohol to excessive energy intake, with the general expectation that alcohol should be associated with positive energy balance not consistently borne out in epidemiological evidence [5, 6, 67]. It is also becoming clear that alcohol interacts with food and macronutrients to influence energy intake [17], but the complexity of these interactions has hindered a clear understanding of the associations. In this study, we took a novel approach, constructing a mechanistic‐ecological model that synthesized established experimental evidence and drew on protein leverage theory to postulate that FGF21 is a key mediator in the relationship between alcohol consumption and food and energy intake, and that dietary pattern is a moderator that influences the magnitude and potentially the sign of the relationship. The model yielded clear predictions, which were strongly supported in our test in an unmanipulated population context.

### 4.1 Testing Predictions in a Population Context

The results of the population analysis aligned closely with the predictions of our model. Alcohol intake was associated with increased consumption of umami‐flavored foods, reduced intake of sweet foods, and decreased carbohydrate intake, as expected from the experimentally established role of FGF21 in increasing protein appetite and decreasing carbohydrate intake. We further observed, as predicted, that the extent to which carbohydrate was displaced in the diets of alcohol consumers by protein vs. fat was dependent on the category of savory foods eaten, high‐fat meats and highly processed savory foods being associated with low‐protein:fat ratios, and minimally processed diets with higher protein:fat ratios. Because the concentration of protein relative to nonprotein macronutrient energy, especially fat [3], is negatively associated with macronutrient energy intake (protein leverage), we predicted the observed spectrum of macronutrient energy intakes among alcohol consumers spanning negative to positive energy balance (Figures 4 and 5).

The present analysis did not measure FGF21. However, evidence from human trials has shown that circulating levels of FGF21 are elevated shortly after consuming alcohol in a dose‐dependent manner [43]. In a human study [43], FGF21 was elevated within 15 min of consuming a bolus of alcohol (25 g) and was significantly elevated at 90 min post‐consumption but was no longer significantly elevated at 4 h post‐consumption of alcohol. Chronic alcohol consumption led to prolonged elevation of FGF21 and participants who drank an average of 22 beers at Oktoberfest had elevated circulating levels of FGF21 for 3 days after drinking [43]. Consistent with our findings, a recent systematic review that examined the effect of alcohol on macronutrient intake found that protein and fat intakes were elevated while carbohydrate intake was reduced after ingesting alcohol [17]. Similarly, animal studies have shown that FGF21 elevates protein‐seeking when alcohol is consumed, while sweet preference is inhibited [31, 32].

An important component of our model through which physiological regulation of appetite control, specifically FGF21, is linked to food environments is the relationship between the flavor and the nutrient content of foods. Recent studies in Australia indicate that most foods can be classified into sweet or savory/salty classes [54]. Savory taste has been shown to correlate positively with the protein content of foods [54, 58], because umami is associated with key amino acids, notably glutamate, either alone or in combination with the ribonucleotide, inosine 5′‐monophosphate, including monosodium glutamate (MSG), which is found in almost all natural sources of protein. However, MSG is also commonly added to ultra‐processed snack foods as a flavor enhancer [68], providing the gustatory experience naturally associated with protein in low‐protein foods (“protein decoys”), and for this reason, the correlation between umami and protein is weaker in highly processed foods [54]. Experiments have shown a tendency to overestimate the protein content of low‐protein processed savory foods common in the modern food supply [55]. Significantly, the global savory ingredients market is rapidly expanding, forecast to reach $13.38 billion in 2032, with MSG anticipated to hold a dominant (55.4%) share [69].

Among the likely commercial reasons for the success of protein decoys is that protein is relatively expensive compared with fats and carbohydrates [70], and that low‐protein macronutrient mixtures (c. 5%) with intermediate carbohydrate and fat are particularly palatable [16, 71]. Protein decoys are not, however, without societal costs. The correlation with umami flavor and actual protein content of foods has been reported to vary across countries, being higher in France (62%) than the Netherlands (54%) and Malaysia (51%), and only 27% in Australia [72]. This is predictive of the pattern of obesity seen in these countries, with Australia experiencing some of the highest rates of obesity in the world at (31.7% of adults) compared to Malaysia (23.1%), France (16.3%) and the Netherlands (15%) [73]. Interestingly, savoriness and salt are more strongly associated with high energy density in foods than is sweetness, and are preferred in obesity more than sweetness [74, 75]. This association potentially provides a set of positive biological and socio‐economic feedbacks in UPF environments, in which umami is associated with energy‐dense foods, obesity predisposes to increased targeting of umami, and increased demand for savory UPF stimulates increased supply.

Our model suggests that alcohol intake could amplify these relationships in food environments replete with savory‐flavored UPFs and high‐fat unprocessed meats. First, an FGF21 associated increase in umami‐seeking would predispose to low‐protein diets with high energy density, a combination that is particularly susceptible to protein leverage [3]. This is consistent with the fact that, in our study, high alcohol consumers on UPF diets were concentrated in the peak region in the macronutrient space for energy intake and had elevated macronutrient and total energy intake compared to nonconsumers (Figure 5). Second, increased protein appetite will result in exacerbated protein leverage (Figure 1B), as suggested in our analysis (Figure 4). FGF21‐mediated increase in protein appetite has been proposed as an explanation for other factors associated with increased risk of obesity. For example, increased umami targeting in obesity (mentioned above) is associated with decreased nitrogen efficiency and increased circulating levels of FGF21 and protein appetite, which has been proposed as a positive feedback loop in obesity [24]. The same mechanism has been hypothesized for elevated risk of obesity in the transition from high‐protein traditional diets to western diets (e.g., Arctic Inuit), early developmental exposure to high‐protein infant formula [63], and hormonally mediated decreased nitrogen efficiency in the menopause transition [77].

The fact that, alongside UPF, high‐fat unprocessed meats were also associated with high energy intakes that were exacerbated in alcohol consumers (Figure 3B) is relevant to the salient question of whether nutrient‐based or processing‐based food classification systems are better suited for public health research and practice. We will not enter that debate here, except to note that this is a complex and important issue, for which there are likely different answers for different questions (e.g., public health research vs. practice) and contexts (e.g., the details of local food systems). In the present study involving a national survey of Australian diets, we contrasted the nutrient‐based Australian food classification system (ADG) and NOVA. The ADG has previously been shown to better predict positive energy balance in Australia than does NOVA, but not in the United States [59]. The present study suggests one reason for this: high‐fat unprocessed meats, which share the key functional property with savory UPF of combining umami flavor with a low‐protein:fat ratio (Figure 3B), are not classified as UPF in the NOVA system but are classified as a “not recommended” food in ADG. In a country such as Australia where the correlation between umami flavor and food protein content is weak (27%), and alcohol intake relatively high [78], it is important to identify all food categories that contribute to this disjunction. On the other hand, the NOVA system has many benefits over nutrient‐based classifications, not least its suitability for food systems analysis and as a policy tool [79], and its resilience to corporate political activity in which nutritional complexity is exploited, for example in misleading marketing [80]. Furthermore, like the present study, a secondary analysis of the NHANES diet surveillance data showed a tight negative correlation across quintiles of percentage energy contributed by UPF and dietary protein density, and a positive relationship with energy intake, while absolute intake of protein remained invariant [27]. That study did not, however, examine the causal contribution of alcohol consumption.

### 4.2 Practical Implications

An important practical question is whether our mechanistic‐ecological model can help inform interventions for improved public health in relation to the role of protein leverage in mediating the interaction of UPF and alcohol in the control of food intake. The model and its testing in a population context suggest that the hyperphagic effects of savory UPFs are exacerbated when consuming alcohol, and that substituting UPF for less processed, lean, diets mitigates this effect. It also raises the topical and important question of whether the same outcome can be achieved through the reformulation of UPF to contain higher protein.

A recent randomized control trial directly tested this. Kwok et al. [81] compared the intake of high protein (HP) vs. standard protein (SP) savory snacks by 19 healthy participants over a 150‐min session that commenced 30 min after drinking white wine (30 g alcohol). The HP and SP treatments consisted of retail‐available solid snacks such as crisps with higher vs. lower protein, combined with a dip that had been fortified with whey isolate powder in HP but not SP. The data are not directly presented, but we calculated that the SP and HP treatment comprised 5% and 16.7% protein, respectively. The short‐term study found no difference in either food mass or energy eaten on the two treatments. This might be interpreted as inconsistent with our model because if protein leverage were a mediator of macronutrient–alcohol interactions, consumption should be lower on the HP treatment.

On closer consideration, however, this is not necessarily the case. Our model predicts that amounts eaten should be determined by the interaction of protein leverage, a homeostatic mechanism, with attraction to umami, a hedonic mechanism. Such interactions between homeostatic and hedonic mechanisms are usual in the regulation of intake, typically with hedonic factors being dominant in the short term but counterbalanced by homeostatic mechanisms in the longer‐term regulation of intake [82]. In the short‐term study of Kwok et al. [81], it is plausible that the leveraging of intake on the low‐protein treatment was counterbalanced by the alcohol‐induced increased palatability of umami on both treatments.

This interpretation is consistent with the results of a second RCT designed to test the effects of protein fortification for mitigating the hyperphagic properties of UPF, but not involving alcohol. Hägele et al. [83] compared responses of 21 healthy adults to ultra‐processed diet (>80% UPF) consisting of 30% vs.13% protein energy and, unlike Kwok et al. [81], observed a 196 Kcal/day (5.7%) reduction in energy intake on the high‐protein treatment, which was not sufficient to restore energy balance, but together with increased energy expenditure (128 Kcal/day) reduced positive energy imbalance from 32% to 18%. Several differences in the design of Kwok et al. vs. Hägele et al. might contribute to the contrasting outcomes. First, in Hägele et al., subjects did not consume alcohol, and therefore the hedonic response to umami would not be accentuated. Second, exposures in Hägele et al. were longer‐term—54 h vs. 150 min in Kwok et al.—providing greater opportunity for homeostatic mechanisms (protein leverage) to influence intake. Third, treatments in Hägele et al. contrasted UPF diets, rather than responses to UPF snack foods by subjects fed a standardized meal 4 h prior. This third contrast is particularly important because it means that subjects in Hägele et al. were tested in a state of cumulative protein imbalance, the same situation for which protein leverage is a homeostatic mitigating response, whereas Kwok et al. compared responses of subjects that entered the trial phase in a similar state of protein balance. In a previous RCT designed to test for protein leverage more generally—i.e., not specifically in protein‐fortified UPF—Gosby et al. [21] found that increased energy intake by subjects confined to low‐protein diets (10% vs. 25%) for 4 days were associated largely with greater intakes of savory snack foods eaten between meals, but in that case the subjects were chronically exposed to low protein.

Experiments to date thus suggest that reformulation of UPF via protein fortification might to some extent mitigate the protein leverage effect on UPF diets, but not entirely, and the efficacy is sensitively dependent on circumstances. However, two recent randomized control trials (not involving alcohol consumption) provide direct experimental evidence for the efficacy of substituting UPF with minimally processed diets in mitigating the hyperphagic effects of protein leverage. In a cross‐over design RCT, Hall et al. [1] found that energy intakes and weight gain in 20 inpatient adults fed ultra‐processed diets for 14 days were higher than when fed minimally processed diets, yet protein intakes were the same. In a similar design, Hamano et al. [2] found that energy intake and weight gain were higher in nine hospital inpatients fed an ultra‐processed diet for a week than when fed a minimally processed diet yet, as in Hall et al., protein intake did not differ. Both studies implicated protein leverage as a mediating mechanism.

### 4.3 Strengths and Limitations

A potential limitation of the study derives from the usual caveats surrounding population surveillance data and estimation of dietary intakes [28, 84, 85]. However, in the present case, the mechanistic premises underlying our predictions—including protein leverage, elevated FGF21 with alcohol consumption, and consequent umami‐seeking and sweet avoidance—have already been established in prior experimental studies. The primary purpose of the study was to construct a model that combines these experimentally verified factors to predict how they might influence energy balance in contrasting ways under different dietary patterns. The core premise, protein leverage, has additionally been validated against spurious associations when analyzing diet surveillance data, using simulations [28]. Additional limitations are that the survey was conducted during 2011–2012, the most recent available survey of a representative sample of the Australian population at the time of analysis [57], and the data have a resolution of 24 h. While we see no reason to expect differences in the biological mechanisms underlying our model, it would be interesting to examine whether relevant subsequent changes in the Australian food environment might bear upon its predictions. A further limitation is that the precise timing of alcohol and food consumption within the 24‐h survey period is not known. However, it is known that alcohol was consumed on the same day that total umami was elevated and sweet foods were decreased relative to respondents who did not report alcohol consumption on the survey day, consistent with the prior experimental data. It is also possible that some people may have consumed large amounts of alcohol prior to but not on the survey day and therefore have chronically elevated FGF21. Despite this possibility, each standard drink reported on the survey day was associated with an increase in umami foods and a decrease of sweet foods. A related consideration is that umami exposure in our analysis is inferred from food categories rather than direct sensory measurement, and savory foods vary in salt, fat, and hedonic appeal independently of protein content. Our model does not, however, assume that umami cues reliably index protein in all food items; rather, it assumes that umami flavor is *more* reliably associated with protein in whole food environments than in food environments in which savory flavoring is engineered independently of protein content, an assumption which is logically and empirically supported (see Introduction).

Among the strengths of the study design are that it tested a priori predictions derived from experimental studies in a large representative population survey, and that 24‐h recalls enable detailed examination of food consumption patterns. We have also applied an integrative approach that combines mechanistic‐ecological models with multidimensional Nutritional Geometry to understand how human regulatory biology interacts with food environments to influence dietary choice and energy intake. More broadly, this approach illustrates the complementarity of experimental and observational methods in nutrition science, demonstrating how experimentally established mechanisms can generate and constrain predictions tested in population contexts [3, 28, 84, 85].

Perhaps the most distinctive contribution of mechanistic‐ecological models of the sort we have constructed is that the same evidence used to build the model including experimentally established mechanisms also constrain interpretation of its predictions, helping to guard against confounding, causal misspecification (including reverse causality), and interpretive overreach. For example, our inability to determine the temporal order of alcohol consumption versus food intake on the survey day introduces the risk of reverse causality—i.e., the possibility that consumption of high umami meals stimulated alcohol intake, rather than vice versa. However, the reverse causal direction would require a signaling loop in which savory intake stimulates alcohol consumption, which has little empirical support. Moreover, even if a tentative mechanistic link were assumed, it would need to account for the full pattern of results—including decreased sweet preference and the interaction with dietary pattern—as does our FGF21‐based model. A second example concerns the general problem of underreporting of UPF and alcohol intake in dietary survey data. However, known underreporting biases—particularly for energy dense foods and alcohol—should attenuate, not artificially inflate, the associations predicted by our model, and therefore the likely impact of underreporting is that our findings underestimate the true effect size and any detectable association is thus conservative. As a third example, we note that several behavioral pathways—including alcohol‐related disinhibition, changes in meal timing, and social eating contexts—can influence short‐term food choice and energy intake. Importantly, however, these pathways do not readily generate the specific directional predictions of our model, nor account for the observed interaction with dietary pattern. Moreover, while such factors undoubtedly contribute to variation in intake, none readily explains the nutrient‐specific pattern—notably the selective increase in savory over sweet foods and its interaction with dietary protein:fat ratio that follows directly from the known mechanistic links between FGF21 and appetite regulation.

An important priority for future research is to extend the model to include the link between energy intake, energy expenditure [86, 87], and macronutrient oxidation rates, which alcohol profoundly affects [88, 89]. Despite their importance for energy balance, we excluded them from our model because the data did not include reliable measures of energy expenditure and retention against which we could test the predictions. Our study nonetheless provides a foundation for a more targeted investigation of the role of macronutrient oxidation in contexts where relevant data are available.

A final consideration concerns the extent to which data collected within a short period (24 h) and in Australia can be generalized more broadly, given that food environments, alcohol consumption patterns, and food product formulations vary substantially across societies. Here, our model explicitly predicts that the effect of alcohol on dietary intake depends on the structure of the local food environment, particularly the degree to which umami flavor is associated with protein content. The purpose of analyzing the Australian dataset was therefore not to estimate a global effect, but to test the directional predictions derived from our mechanistic model within a specific, well‐characterized environment: in food environments where umami flavor is frequently associated with protein‐dilute UPFs, alcohol‐driven increases in savory/protein appetite should lead to higher energy intake. Australia, like many high‐income countries, combines high UPF consumption with strong umami‐protein decoupling and high alcohol intake, making it an appropriate system to test this prediction. Crucially, the validity of our mechanistic inference does not depend on the assumption that Australia's food environment is representative of all societies—rather, our study illustrates how the model behaves in one well‐characterized context, and the predictions it generates are explicitly conditional on the structure of the food environment. How the model performs in other contexts remains an open and important question, and we hope our study provides a framework for replication elsewhere.

## 5 Conclusions

Our study introduces an integrated modeling approach and applies it to examine how alcohol can interact with dietary patterns to explain contrasting results in studies examining the effects of its consumption on energy intake. Results suggest that alcohol may have contributed to the obesity epidemic by amplifying the hyperphagic effects of obesogenic food environments, particularly those that oversupply low‐protein savory foods in the context of habitual alcohol intake. Advice to limit highly processed foods, including savory snack foods, may be even more critical than previously thought for reducing the risk of weight gain among adults who drink regularly. Protein fortification of UPF might, at best, partially mitigate the interaction of protein leverage with alcohol consumption, but further experimental studies are needed to test this. Our findings are, however, consistent with the view that substitution of UPF for minimally processed foods would attenuate this interaction. Additionally, more attention should be paid in the setting of dietary guidelines to unprocessed umami‐flavored foods rich in fat, such as high‐fat meats, consumption of which might be elevated in the context of alcohol drinking.

We believe that mechanistic‐ecological models have broader theoretical and methodological implications for nutrition science, through providing a principled and structured mechanism for integrating evidence from causally tight experimental studies with ecologically relevant observational population data. For example, we cannot claim that our observational analysis provides direct evidence for the causal role of FGF21, and neither is this its role. Rather, in our study, the causal efficacy of FGF21 comes from previous experimental work, where its effects on macronutrient appetites and flavor preferences are well characterized. We have treated these findings as inputs to a mechanistic model that generates explicit, testable predictions about how alcohol consumption should influence diet composition in real‐world settings. The role of population data is explicitly not to establish causality, but to test whether the mechanistic structure of the model is relevant in more complex, real‐world settings. Population data do, however, also provide secondary evidence bearing on the causal plausibility of the model—if the mechanism is valid, then the patterns predicted by that mechanism should be detected in independent observational data, even though the mechanism is not measured directly. This triangulation approach—combining experimental causal inference with ecological validation—offers a causally anchored framework for nutrition science more broadly.

## Author Contributions

D.R., A.G., and S.J.S. contributed to the study's conception and design; A.G. conducted the analysis; D.R. led the theoretical framing, with contributions from A.G. and S.J.S.; A.G. and D.R. drafted the manuscript, and D.R., A.G., and S.J.S. revised.

## Funding

The National Health and Medical Research Council, Australia, Nutrition and Complexity Program Grant, grant/award number GNT1149976.

## Conflicts of Interest

The authors declare no conflicts of interest.

## Supporting information


**Data S1:** Supplementary information.

## Data Availability

The data that support the findings of this study are available on request from the corresponding author. The data are not publicly available due to privacy or ethical restrictions.
