# Supplementary material for: Protein Decoys, Alcohol, and Energy Intake: Testing a Mechanistic‐Ecological Model
Source: Obes Rev. 2026 May 19;27(10):e70138. doi: 10.1111/obr.70138 (PMC13551414; doi:10.1111/obr.70138)
Supplement: Supplementary file 1 [file OBR-27-e70138-s001.pdf]

Ultra-processed protein decoys, alcohol, and energy intake: testing a mechanistic-ecological model

Amanda Grech [a,b,*], Stephen J. Simpson [a,b], David Raubenheimer [a,b,*]

[a] Charles Perkins Centre, University of Sydney, NSW, Australia, 2006.

[b] School of Life and Environmental Science, University of Sydney, Australia, 2006

Keywords: protein | alcohol | energy balance

Running head: Alcohol: a mechanistic-ecological model

To whom correspondence may be addressed.

* David Raubenheimer. Address: Charles Perkins Centre, University of Sydney, NSW, Australia, 2006. Telephone number: +61 2 9351 3242. Email: david.raubenheimer@sydney.edu.au

*Amanda Grech. Address: Charles Perkins Centre, University of Sydney, NSW, Australia, 2006. Telephone number: +61 2 8627 9446. Email: amanda.grech@sydney.edu.au

Supplementary Table S1: Characteristics of all consumers (n= 3,239) and non-consumers (n= 6,193) of alcohol from the National Nutrition and Physical Activity Survey.

Characteristics	Consumers	(SE)	Non-consumers	(SE)	P-Value
**Gender (% male)**	59.2	(0.9)	44.3	(0.5)	<.0001
**Age (%)**					
18-50	52.6	(0.8)	62.3	(0.4)	
51-70	35.2	(0.7)	26.5	(0.3)	
71+	12.2	(0.6)	11.2	(0.3)	<.0001
**Country of Birth (%)**					
Australia	73.0	(1.1)	67.3	(1.0)	
Canada, Ireland, NZ, South Africa, UK, USA	14.0	(0.8)	10.1	(0.5)	
Other	13.0	(0.9)	22.6	(0.9)	<.0001
**SEIFA (%)**					
Lowest – Quintile 1	15.4	(1.2)	19.5	(1.1)	
Middle – Quintile 2-3	56.8	(1.6)	61.0	(1.5)	
Highest – Quintile 5	27.8	(1.2)	19.5	(1.0)	<.0001
**Tertiary Education (%)**					
No Tertiary education	34.3	(1.1)	43.3	(0.8)	
Vocational College	38.5	(1.1)	33.0	(0.8)	
University	27.2	(1.0)	23.8	(0.8)	<.0001
**Body Mass Index**					
Underweight (<18.5 kg/m2)	1.1	(0.2)	2.4	(0.3)	
Normal (≥18.5 - <25 kg/m2)	33.4	(1.1)	37.5	(1.0)	
Overweight (≥25.0 - <30.0 kg/m2)	42.2	(1.2)	32.9	(0.9)	
Obese (≥30.0 kg/m2)	23.3	(1.0)	27.3	(0.8)	<.0001
**Geographic Area**					
Major cities of Australia	70.6	(1.2)	71.9	(0.7)	
Inner regional Australia	19.9	(1.1)	18.7	(1.0)	
Other	9.5	(0.9)	9.3	(0.9)	0.6114
**Macronutrient Intake**					
Alcohol (g)	42.5	(0.9)	0.0	0.0	<.0001
Energy (kJ)	9901.3	(82.7)	8065.7	62.7	<.0001
Protein (g)	97.9	(1.0)	87.8	0.8	<.0001
Carbohydrate (g)	229.7	(2.4)	224.7	1.9	0.1089
Fat (g)	80.7	(1.1)	71.1	0.7	<.0001

SEIFA, socio-economic index for area

P-value, chi-square for categorical variables, students t-test for continuous variables

Supplementary Table S2: Coefficients from mixture models for alcohol (mL) for alcohol consumers

Model	AIC	Coefficients (Coef.)
1	35698.77	SEIFA
2	35694.35	Age
3	35668.91	Sex
4	33108.75	p_P + p_F + p_C + SEIFA + Age + Sex
5	33114.13	p_P + p_F + p_C + p_P:p_C + p_P:p_F + p_F +:p_C + SEIFA + Age + Sex

Model 4 favoured by AIC.

Coef.	Estimate	Std. Error	t value	df	p
p_P	69.9267	12.1118	5.7734	3133	0
p_F	66.0572	7.1565	9.2303	3133	0
p_C	36.1618	4.7041	7.6873	3133	0
SEIFA	-1.6755	0.8448	-1.9833	3133	0.0474
Age	-3.2844	0.8457	-3.8839	3133	1.00E-04
Sex	7.1024	0.8462	8.3936	3133	0

p_P: Proportion of energy from protein; p_F: proportion of energy from fat; p_C: proportion of energy from carbohydrate; SEIFA: socio-economic index for area

Supplementary Table S3: Coefficients from mixture models for total savory food intake (g) for alcohol consumers

Model	AIC	Coefficients (Coef.)
1	45323.61	SEIFA
2	45304.72	Age
3	45263.71	Sex
4	40800.24	p_P + p_F + p_C + SEIFA + Age + Sex
5	40802.57	p_P + p_F + p_C + p_P:p_C + p_P:p_F + p_F +:p_C + SEIFA + Age + Sex

Model 4 favoured by AIC.

Coef.	Estimate	Std. Error	t value	df	p
p_P	847.9142	41.2366	20.5622	3133	0
p_F	520.2736	24.3657	21.3527	3133	0
p_C	-146.486	16.0159	-9.1462	3133	0
SEIFA	-9.7323	2.8764	-3.3835	3133	7.00E-04
Age	-31.4115	2.8792	-10.9099	3133	0
Sex	47.4427	2.8809	16.4679	3133	0

p_P: Proportion of energy from protein; p_F: proportion of energy from fat; p_C: proportion of energy from carbohydrate; SEIFA: socio-economic index for area

Supplementary Table S4: Coefficients from mixture models for discretionary sweet food intake (g) for alcohol consumers

Model	AIC	Coefficients (Coef.)
1	50381.89	SEIFA
2	50278.97	Age
3	50349.35	Sex
4	48550.58	p_P + p_F + p_C + SEIFA + Age + Sex
5	48511.33	p_P + p_F + p_C + p_P:p_C + p_P:p_F + p_F +:p_C + SEIFA + Age + Sex

Model 5 favoured by AIC.

Coef.	Estimate	Std. Error	t value	df	p
p_P	4836.557	1246.601	3.8798	3130	1.00E-04
p_F	123.9154	474.9448	0.2609	3130	0.7942
p_C	2170.987	221.5314	9.7999	3130	0
SEIFA	-22.763	9.8239	-2.3171	3130	0.0206
Age	-118.509	9.8469	-12.0351	3130	0
Sex	71.0081	9.8357	7.2194	3130	0
p_P:p_C	-13034.9	2053.212	-6.3485	3130	0
p_P:p_F	-5726.28	2672.094	-2.143	3130	0.0322
p_F:p_C	-155.745	1002.971	-0.1553	3130	0.8766

p_P: Proportion of energy from protein; p_F: proportion of energy from fat; p_C: proportion of energy from carbohydrate; SEIFA: socio-economic index for area

Supplementary Table S5: Coefficients from mixture models for savory UPF (g) for alcohol consumers

Model	AIC	Coefficients (Coef.)
1	54841.34	SEIFA
2	54776.6	Age
3	54792.46	Sex
4	52972.14	p_P + p_F + p_C + SEIFA + Age + Sex
5	52964.53	p_P + p_F + p_C + p_P:p_C + p_P:p_F + p_F +:p_C + SEIFA + Age + Sex

Model 5 favoured by AIC.

Coef.	Estimate	Std. Error	t value	df	p
p_P	3516.461	2533.886	1.3878	3130	0.1653
p_F	2746.407	965.3902	2.8449	3130	0.0045
p_C	-61.1261	450.2928	-0.1357	3130	0.892
SEIFA	-47.9187	19.9684	-2.3997	3130	0.0165
Age	-203.499	20.0152	-10.1673	3130	0
Sex	197.6093	19.9924	9.8842	3130	0
p_P:p_C	-8793.96	4173.435	-2.1071	3130	0.0352
p_P:p_F	-10239	5431.396	-1.8852	3130	0.0595
p_F:p_C	5189.718	2038.676	2.5456	3130	0.011

p_P: Proportion of energy from protein; p_F: proportion of energy from fat; p_C: proportion of energy from carbohydrate; SEIFA: socio-economic index for area

Supplementary Table S6: Coefficients from mixture models for high unprocessed fat meat (>10 g fat/100 g) (g) for alcohol consumers

Model	AIC	Coefficients (Coef.)
1	51120.4	SEIFA
2	51119.95	Age
3	51089.07	Sex
4	50060.8	p_P + p_F + p_C + SEIFA + Age + Sex
5	49900.9	p_P + p_F + p_C + p_P:p_C + p_P:p_F + p_F +:p_C + SEIFA + Age + Sex

Model 5 favoured by AIC.

Coef.	Estimate	Std. Error	t value	df	p
p_P	-3093.15	1555.441	-1.9886	3130	0.0468
p_F	6375.21	592.6104	10.7578	3130	0
p_C	1469.033	276.4149	5.3146	3130	0
SEIFA	-1.2159	12.2577	-0.0992	3130	0.921
Age	-22.5366	12.2864	-1.8343	3130	0.0667
Sex	97.4288	12.2724	7.9388	3130	0
p_P:p_C	3105.288	2561.887	1.2121	3130	0.2256
p_P:p_F	2460.46	3334.094	0.738	3130	0.4606
p_F:p_C	-15360.5	1251.453	-12.2741	3130	0

p_P: Proportion of energy from protein; p_F: proportion of energy from fat; p_C: proportion of energy from carbohydrate; SEIFA: socio-economic index for area

Supplementary Table S7: Coefficients from mixture models for not recommended savory food (g) (ADG definition) for alcohol consumers

Model	AIC	Coefficients (Coef.)
1	56956.77	SEIFA
2	56928.54	Age
3	56883.55	Sex
4	54663.6	p_P + p_F + p_C + SEIFA + Age + Sex
5	54648.56	p_P + p_F + p_C + p_P:p_C + p_P:p_F + p_F +:p_C + SEIFA + Age + Sex

Model 5 favoured by AIC.

Coef.	Estimate	Std. Error	t value	df	p
p_P	58.4103	3313.473	0.0176	3130	0.9859
p_F	10538.4	1262.407	8.3479	3130	0
p_C	1299.962	588.8319	2.2077	3130	0.0273
SEIFA	-82.0699	26.112	-3.143	3130	0.0017
Age	-210.194	26.1731	-8.0309	3130	0
Sex	353.9511	26.1433	13.5389	3130	0
p_P:p_C	-6481.75	5457.453	-1.1877	3130	0.235
p_P:p_F	-8120.72	7102.444	-1.1434	3130	0.253
p_F:p_C	-11482.7	2665.904	-4.3072	3130	0

p_P: Proportion of energy from protein; p_F: proportion of energy from fat; p_C: proportion of energy from carbohydrate; SEIFA: socio-economic index for area

Supplementary Table S8: Coefficients from mixture models for recommended savoury foods (g) (lean meat, poultry, eggs, tofu, legumes, nuts and seeds) for alcohol consumers

Model	AIC	Coefficients (Coef.)
1	42730.32	SEIFA
2	42726.64	Age
3	42706.08	Sex
4	38869.09	p_P + p_F + p_C + SEIFA + Age + Sex
5	38872.96	p_P + p_F + p_C + p_P:p_C + p_P:p_F + p_F +:p_C + SEIFA + Age + Sex

Model 4 favoured by AIC.

Coef.	Estimate	Std. Error	t value	df	p
p_P	1215.066	30.3174	40.0782	3133	0
p_F	19.1306	17.9138	1.0679	3133	0.2856
p_C	-174.485	11.775	-14.8182	3133	0
SEIFA	-2.4553	2.1148	-1.161	3133	0.2457
Age	-13.4879	2.1168	-6.3718	3133	0
Sex	19.0568	2.1181	8.9973	3133	0

p_P: Proportion of energy from protein; p_F: proportion of energy from fat; p_C: proportion of energy from carbohydrate; SEIFA: socio-economic index for area

Supplementary Table S9: Coefficients from mixture models total energy (kJ) for non-alcohol consumers

Model	AIC	Coefficients (Coef.)
1	125889.5	SEIFA
2	125859.9	Age
3	125813.9	Sex
4	113281	p_P + p_F + p_C + SEIFA + Age + Sex
5	113242.2	p_P + p_F + p_C + p_P:p_C + p_P:p_F + p_F +:p_C + SEIFA + Age + Sex

Model 5 favoured by AIC.

Coef.	Estimate	Std. Error	t value	df	p
p_P	-2147.78	4267.077	-0.5033	5999	0.6147
p_F	4749.409	1922.037	2.471	5999	0.0135
p_C	3809.731	804.3627	4.7363	5999	0
SEIFA	98.0177	38.751	2.5294	5999	0.0115
Age	-551.372	38.8309	-14.1993	5999	0
Sex	962.6507	38.69	24.8811	5999	0
p_P:p_C	2863.724	7121.459	0.4021	5999	0.6876
p_P:p_F	10371.23	9519.583	1.0895	5999	0.276
p_F:p_C	26921.61	4086.343	6.5882	5999	0

p_P: Proportion of energy from protein; p_F: proportion of energy from fat; p_C: proportion of energy from carbohydrate; SEIFA: socio-economic index for area

Supplementary Table S10: Coefficients from mixture models for carbohydrate, fat and protein energy (kJ) for alcohol consumers

Model	AIC	Coefficients (Coef.)
1	66188.06	SEIFA
2	66164.96	Age
3	66149.73	Sex
4	59210.12	p_P + p_F + p_C + SEIFA + Age + Sex
5	59207.48	p_P + p_F + p_C + p_P:p_C + p_P:p_F + p_F +:p_C + SEIFA + Age + Sex

Model 5 favoured by AIC.

Coef.	Estimate	Std. Error	t value	df	p
p_P	8449.779	6849.471	1.2336	3130	0.2174
p_F	16951.09	2609.593	6.4957	3130	0
p_C	6113.298	1217.208	5.0224	3130	0
SEIFA	-15.7801	53.9775	-0.2923	3130	0.77
Age	-709.033	54.104	-13.105	3130	0
Sex	1026.253	54.0424	18.9898	3130	0
p_P:p_C	-11563.3	11281.41	-1.025	3130	0.3054
p_P:p_F	-29113.9	14681.87	-1.983	3130	0.0475
p_F:p_C	8256.403	5510.844	1.4982	3130	0.1342

p_P: Proportion of energy from protein; p_F: proportion of energy from fat; p_C: proportion of energy from carbohydrate; SEIFA: socio-economic index for area

Supplementary Table S11: Coefficients from mixture models for total energy including alcohol (kJ) for alcohol consumers

Model	AIC	Coefficients (Coef.)
1	66971.78	SEIFA
2	66950.32	Age
3	66931.55	Sex
4	59648.76	p_P + p_F + p_C + SEIFA + Age + Sex
5	59647.03	p_P + p_F + p_C + p_P:p_C + p_P:p_F + p_F +:p_C + SEIFA + Age + Sex

Model 4 selected.

Coef.	Estimate	Std. Error	t value	df	p
p_P	-2651.01	830.1776	-3.1933	3133	0.0014
p_F	17997.2	490.5309	36.6892	3133	0
p_C	8973.515	322.4336	27.8306	3133	0
SEIFA	-53.4419	57.9083	-0.9229	3133	0.3561
Age	-785.073	57.9639	-13.5442	3133	0
Sex	1188.309	57.9988	20.4885	3133	0

p_P: Proportion of energy from protein; p_F: proportion of energy from fat; p_C: proportion of energy from carbohydrate; SEIFA: socio-economic index for area

Model 5

Coef.	Estimate	Std. Error	t value	df	p
p_P	9583.46	7346.222	1.3045	3130	0.1921
p_F	18973.93	2798.852	6.7792	3130	0
p_C	6791.082	1305.485	5.202	3130	0
SEIFA	-54.4385	57.8922	-0.9403	3130	0.3471
Age	-784.827	58.0278	-13.525	3130	0
Sex	1188.654	57.9618	20.5075	3130	0
p_P:p_C	-9664.86	12099.59	-0.7988	3130	0.4245
p_P:p_F	-30248.3	15746.66	-1.9209	3130	0.0548
p_F:p_C	7544.583	5910.513	1.2765	3130	0.2019

Supplementary Table S12: Coefficients from mixture models for savoury UPF food (kJ) for alcohol consumers

Model	AIC	Coefficients (Coef.)
1	54841.34	SEIFA
2	54776.6	Age
3	54792.46	Sex
4	52972.14	p_P + p_F + p_C + SEIFA + Age + Sex
5	52964.53	p_P + p_F + p_C + p_P:p_C + p_P:p_F + p_F +:p_C + SEIFA + Age + Sex

Model 5 favoured by AIC.

Coef.	Estimate	Std. Error	t value	df	p
p_P	3516.461	2533.886	1.3878	3130	0.1653
p_F	2746.407	965.3902	2.8449	3130	0.0045
p_C	-61.1261	450.2928	-0.1357	3130	0.892
SEIFA	-47.9187	19.9684	-2.3997	3130	0.0165
Age	-203.499	20.0152	-10.1673	3130	0
Sex	197.6093	19.9924	9.8842	3130	0
p_P:p_C	-8793.96	4173.435	-2.1071	3130	0.0352
p_P:p_F	-10239	5431.396	-1.8852	3130	0.0595
p_F:p_C	5189.718	2038.676	2.5456	3130	0.011

p_P: Proportion of energy from protein; p_F: proportion of energy from fat; p_C: proportion of energy from carbohydrate; SEIFA: socio-economic index for area

Supplementary Table S13: Coefficients from mixture models for high fat unprocessed meat (kJ) for alcohol consumers

Model	AIC	Coefficients (Coef.)
1	51120.4	SEIFA
2	51119.95	Age
3	51089.07	Sex
4	50060.8	p_P + p_F + p_C + SEIFA + Age + Sex
5	49900.9	p_P + p_F + p_C + p_P:p_C + p_P:p_F + p_F +:p_C + SEIFA + Age + Sex

Model 5 favoured by AIC.

Coef.	Estimate	Std. Error	t value	df	p
p_P	-3093.15	1555.441	-1.9886	3130	0.0468
p_F	6375.21	592.6104	10.7578	3130	0
p_C	1469.033	276.4149	5.3146	3130	0
SEIFA	-1.2159	12.2577	-0.0992	3130	0.921
Age	-22.5366	12.2864	-1.8343	3130	0.0667
Sex	97.4288	12.2724	7.9388	3130	0
p_P:p_C	3105.288	2561.887	1.2121	3130	0.2256
p_P:p_F	2460.46	3334.094	0.738	3130	0.4606
p_F:p_C	-15360.5	1251.453	-12.2741	3130	0

p_P: Proportion of energy from protein; p_F: proportion of energy from fat; p_C: proportion of energy from carbohydrate; SEIFA: socio-economic index for area

Supplementary Table S14: Coefficients from mixture models for not recommended savory food (ADG definition) (kJ) for alcohol consumers

Model	AIC	Coefficients (Coef.)
1	56956.77	SEIFA
2	56928.54	Age
3	56883.55	Sex
4	54663.6	p_P + p_F + p_C + SEIFA + Age + Sex
5	54648.56	p_P + p_F + p_C + p_P:p_C + p_P:p_F + p_F +:p_C + SEIFA + Age + Sex

Model 5 favoured by AIC.

Coef.	Estimate	Std. Error	t value	df	p
p_P	58.4103	3313.473	0.0176	3130	0.9859
p_F	10538.4	1262.407	8.3479	3130	0
p_C	1299.962	588.8319	2.2077	3130	0.0273
SEIFA	-82.0699	26.112	-3.143	3130	0.0017
Age	-210.194	26.1731	-8.0309	3130	0
Sex	353.9511	26.1433	13.5389	3130	0
p_P:p_C	-6481.75	5457.453	-1.1877	3130	0.235
p_P:p_F	-8120.72	7102.444	-1.1434	3130	0.253
p_F:p_C	-11482.7	2665.904	-4.3072	3130	0

p_P: Proportion of energy from protein; p_F: proportion of energy from fat; p_C: proportion of energy from carbohydrate; SEIFA: socio-economic index for area

Supplementary Table S15: Coefficients from mixture models for recommend savory food (lean meat, poultry, eggs, tofu, legumes, nuts and seeds) (kJ) for alcohol consumers

Model	AIC	Coefficients (Coef.)
1	54139.35	SEIFA
2	54138.59	Age
3	54116.07	Sex
4	50450.58	p_P + p_F + p_C + SEIFA + Age + Sex
5	50453.21	p_P + p_F + p_C + p_P:p_C + p_P:p_F + p_F +:p_C + SEIFA + Age + Sex

Model 4 favoured by AIC.

Coef.	Estimate	Std. Error	t value	df	p
p_P	6687.112	191.808	34.8636	3133	0
p_F	652.4266	113.3345	5.7566	3133	0
p_C	-1121.06	74.4965	-15.0484	3133	0
SEIFA	-11.3748	13.3794	-0.8502	3133	0.3953
Age	-55.8631	13.3922	-4.1713	3133	0
Sex	119.063	13.4003	8.8851	3133	0

p_P: Proportion of energy from protein; p_F: proportion of energy from fat; p_C: proportion of energy from carbohydrate; SEIFA: socio-economic index for area
